# How integrins control breast biology^☆^

**DOI:** 10.1016/j.ceb.2013.06.010

**Published:** 2013-10

**Authors:** Marina A Glukhova, Charles H Streuli

**Affiliations:** Wellcome Trust Centre for Cell-Matrix Research, University of Manchester, Oxford Road, Manchester M13 9PT, United Kingdom

## Abstract

This article explores new ideas about how the ECM-integrin axis controls normal and malignant breast biology. We discuss the role of integrins in mammary stem cells, and how cell–matrix interactions regulate ductal and alveolar development and function. We also examine the contribution of integrins to tissue disorganisation and metastasis, and how an altered stromal and ECM tumour microenvironment affects the cancer cell niche both within primary tumours and at distant sites. Finally, we mention novel strategies for integrin-directed breast cancer treatment.

**Current Opinion in Cell Biology** 2013, **25**:633–641

This review comes from a themed issue on **Cell adhesion and migration**

Edited by **Carole A Parent** and **Orion D Weiner**

For a complete overview see the Issue and the Editorial

Available online 22nd July 2013

0955-0674/$ – see front matter, © 2013 The Authors. Published by Elsevier Ltd. All rights reserved.

**http://dx.doi.org/10.1016/j.ceb.2013.06.010**

## Introduction

**Figure 1:**
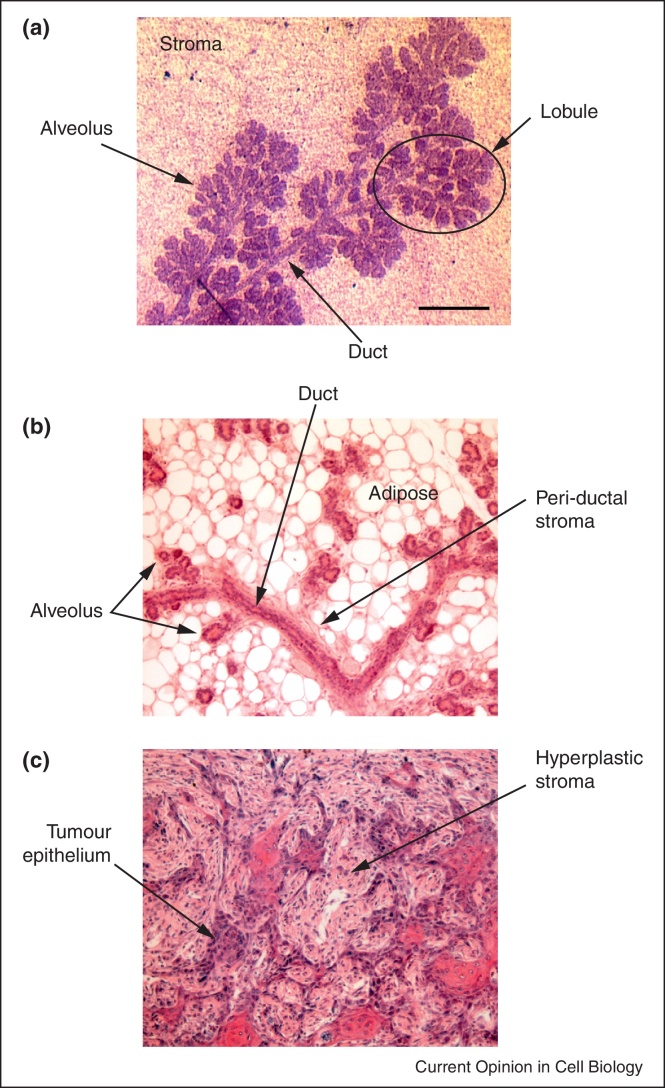
**(a)** Mammary gland from 14.5-day-pregnant mouse stained in whole-mount with Carmine-Alum, showing ducts and lobules embedded within connective tissue stroma. **(b)** A section through a mammary gland from 14.5-day-pregnant mouse stained with H&E, showing the discrete organisation of epithelial alveoli and ducts surrounded by the stromal ECM. The whole epithelial structure is embedded within the connective tissue stroma, largely containing adipocytes in the mouse. **(c)** A section through an invasive mammary tumour developed in K5ΔNβcat mouse (courtesy of Aurélie Chiche). Note that the tissue architecture is completely disrupted. Bars: (a) 0.5 mm, (b,c) 0.2 mm.

Building and maintaining epithelial tissues is a complex process. Even in a relatively simple organ such as mammary gland, the formation of a network of ducts and associated alveoli requires sophisticated interactions between epithelium and the surrounding microenvironment (Figure 1a,b). Ductal and alveolar breast epithelia adhere to the type of ECM known as basement membranes (BM). Stromal cells bind directly to interstitial ECM. Both local epithelial cell–ECM interactions and long-range communication between epithelium and stroma are essential for all aspects of normal mammary gland development and function [1–3].

The main ECM receptors of mammary epithelium are integrins, which link ECM with the cytoskeleton and signalling pathways and thereby establish an ECM-integrin-signalling axis. Integrins function as microenvironmental sensors that control cell phenotype and fate decisions [4]. They are on-off switches and rheostats, which modulate cellular responses to growth factor (GF) signals and cytokines, and convert tension forces generated within the microenvironment into intracellular chemical signals [5,6].

The prominence of integrins predicts that they will have defining roles in mammary gland biology and disease. Genetic deletion experiments reveal that integrins control nearly every aspect of mammary gland function [1,3]. Pathological and genetic studies implicate integrins in both early breast cancer and malignant disease [7^•^,8,9]. The central role of stromal cells in mammary development and cancer also predicts key roles for integrins in the fibroblast, adipocyte and macrophage compartment of breast. However, genetic analyses have yet to explore integrin function in stromal cell types.

In this review, we focus on new ideas about how the ECM-integrin axis controls normal and malignant breast biology.

## Integrins have a central role in the mammary stem cell niche

Mammary ducts are pseudo-stratified epithelia consisting of basal myoepithelial cells that contact ECM, and luminal epithelia that line the ductal lumens [1]. *In vivo* lineage tracing studies suggest that myoepithelial and luminal stem cells diverge at birth, and once formed they do not transition between each other [10]. Integrins are key receptors to maintain mammary stem cells within their niches. Deletion of β1-integrins from luminal cells prevents transplanted mammary epithelial fragments from forming new glands [11]. Genetic removal of β1-integrins specifically within basal myoepithelial cells affects stem cell renewal. Myoepithelial cells normally divide parallel to the plane of the BM, but in the absence of β1-integrins mitotic cells within the basal layer align their spindle poles randomly, leading to a perturbation of cell lineages and epithelial homeostasis [12^••^].

As microenvironmental sensors, integrins provide a mechanistic link between the stem cell niche and stem/progenitor cell fates. The β1 and β3-integrin subunits, as well as the β1 partner α2-integrin, and the β1/β4 partner α6-integrin, are used to isolate stem and progenitor cells, though their individual roles in normal mammary stem cell biology are not yet known [13,14]. In cancer, α6 and β3 integrins are expressed in tumour-initiating cells (TICs) and promote their self-renewal [15,16^•^,17]. For β3-integrin at least, the mechanism is through cooperation with TGFβ [16^•^]. However integrins may have an additional and unique function in mammary stem cells. Placing mammary epithelia in suspension induces virtually all of the cells to undergo anoikis, because they require integrin signalling to prevent Bax-mediated death [18,19^••^]. In ‘mammosphere’ stem cell assays performed with cell suspensions, floating stem cells uniquely survive and proliferate to form mammospheres, which can regenerate epithelial ducts/alveoli if implanted into mammary fat pads [20].

The adhesion signalling components of the ECM-integrin axis, which provide normal mammary stem cells with the capacity of anchorage-independent growth, remain to be defined. In the Polyoma-Middle T breast cancer model, focal adhesion kinase (FAK) maintains TICs, and genetic deletion of Fak impairs tumorigenicity [21]. Integrins and GFs also co-stimulate signalling pathways to maintain stem cells. In TICs of triple-negative breast cancer, neuropilin-2 collaborates with α6β1 integrin to activate the Fak/Ras/Mek pathway and the expression of the Hedgehog effector Gli1. In turn, Gli1 induces the expression of both the stem cell regulator BMI-1 and neuropilin-2, thereby creating an autocrine loop to maintain the stem cell niche [22,23^•^].

Understanding the mechanisms linking integrin-mediated recognition of the stem cell niche with the profile of transcription factors that determine the identity of both normal breast stem cells and TICs is a pressing area for the future.

## Cell–matrix interactions and integrins are essential for ductal morphogenesis

The ductal architecture of mammary gland is formed by unique mini-organs called terminal endbuds, which invade stroma and bifurcate to produce a branching network of ducts [24]. Endbuds are surrounded by BM and require β1-integrins to generate motility [25]. 3D models, where duct-like structures form and elongate through ECM (i.e. Matrigel) in response to stromal GFs such as FGF2, are providing novel insights into the ductal morphogenesis programme [26]. Cells within cultured ‘endbuds’ do not extend lamellipodia in the way that migrating cells on 2D substrata do, rather the cells have smooth edges and appear to shuffle together through the ECM [27^•^]. Although β1-integrins are required for cultured ducts to form (Streuli lab, unpublished data), the mechanisms of integrin-dependent migration remain to be defined.

Little is known about the processes controlling branching of mammary ducts. In prepubertal gland, collagen I fibers are oriented towards the long axis of the mammary fat pad, and upon puberty, the branching epithelium follows pre-existing collagen tracks suggesting that the stroma provides spatial cues to direct branch growth [28^••^]. Salivary gland is related to breast, and although mammary and salivary differentiation programmes are cell autonomous, ductal patterning is controlled directly by the stroma of the host tissue [29]. This further argues that cell–matrix interactions with stromally derived ECM proteins influence branch patterns. Indeed, fibronectin assembly and the fibronectin-induced regulator Btbd7, local accumulation of tenascin, MT1-MMP, and NC1 domains of collagen-IV, as well as integrin-mediated ROCK1-myosin II, FAK and ERK signalling, all have important roles in glandular branching morphogenesis [29,30,31,32,33^•^].

The new imaging systems to study how ducts form, and discoveries about the GFs and ECM proteins involved, promise to offer far-reaching insights into how normal epithelial morphogenesis occurs and how it is subverted during malignancy [34^•^,35].

## Integrins control the differentiated function of mammary alveoli and lumen formation

**Figure 2:**
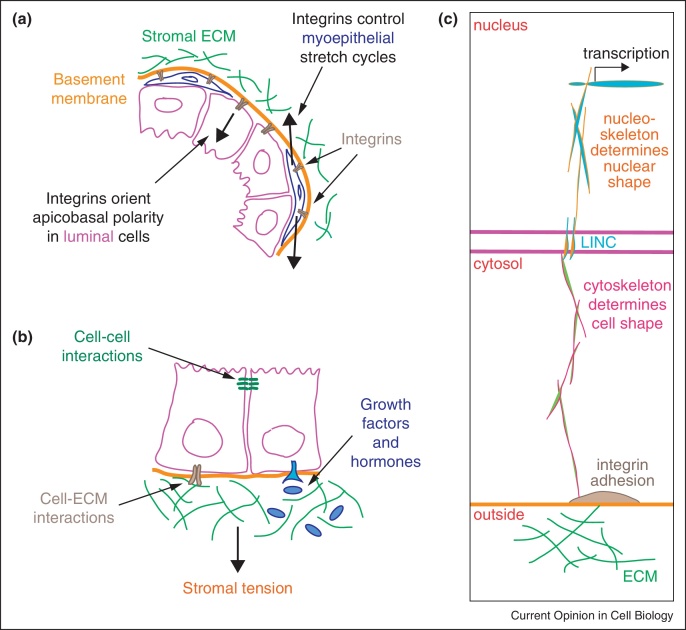
**(a)** Diagram of a part mammary alveolus showing key functional integrin-dependent forces identified by genetic analyses. β1-integrins are required for luminal cells to orientate their polarity so that they can secrete milk apically into lumens, and for myoepithelial cells to complete the stretch-relax cycles needed for milk ejection. **(b)** Epithelial cell function depends on the integration a variety of microenvironmental signals, including those from GFs, cell–ECM and cell–cell interactions, and from biomechanical forces. **(c)** Cell–ECM interactions can regulate transcription and cell phenotype directly by a series of mechanical links between ECM, acto-myosin cytoskeleton, the LINC nuclear membrane complex, and nuclear cytoskeleton.

By contrast to the elongated nature of ducts, lactational alveoli are roughly spherical. Clusters of alveoli gather in terminal ductal lobular units (Figure 1a). Alveoli contain a single layer of polarised luminal epithelial cells surrounded by sparse myoepithelia — both cell types contact the basally located BM. Alveoli produce milk and secrete it apically into the luminal space, from where it is squeezed into ducts by the contraction of myoepithelial cells. Genetic analyses reveal that luminal polarity and myoepithelial contraction both require β1-integrins [36^••^,37^••^] (Figure 2a).

Interactions between laminin and integrins orient the microtubule network into an apical-basal direction by recruiting microtubule plus tips via ILK and EB1 [37^••^]. This triggers endocytosis of apical membrane components, leading to the formation of an apical surface at the opposite side of the cell where the lumen forms. It is not yet known how integrins organise microtubules, but possibilities include an ILK-mediated link to IQ-GAP and Dia1 [38] and/or interactions between laminin-binding integrins, the PH-domain protein LL5, and the microtubule plus-tip binding protein, Clasp [39^•^]. Microtubules may also dynamically regulate adhesion via the MT motor protein KIF14, which controls inside-out integrin activation via Rapil and Rap1 [40^•^].

Once made, milk is ejected from the alveolar lumens into ducts in response to oxytocin-induced myoepithelial contraction. The contractile force generated within cells needs an equal and opposite force from the surrounding ECM, which is transmitted through integrins. In the contraction-relaxation cycle, oxytocin induces contraction via a Rho/Rock/myosin tension pathway, while α3β1-integrin relaxes tension via Fak/Rac/Pak signalling to inhibit MLCK [36^••^] (Figure 2a).

Integrins and tension also control gene expression in luminal cells. The BM provides essential instructive signals for mammary differentiation via β1-integrins and specific components of adhesion complexes [4]. These signals guide tissue-specific gene expression in conjunction with temporal cues from the cytokine, prolactin [41–45]. In a separate mechanism, the elasticity of the local microenvironment and its effects on intracellular tension determine the levels of prolactin receptor — tension blocks transcription of the receptor [46^•^]. Tissue-specific gene expression is therefore controlled not only by biochemical signals, that is, soluble factors and ECM, but also by mechanical forces [47].

Thus, converging signals from the cellular microenvironment, comprising hormones, ECM proteins and stromal tension coordinate alveolar cell function (Figure 2b). Hormones and ECM respectively provide temporal and spatial cues to activate milk synthesis in the appropriate luminal cells, while perhaps expansive forces antagonise endocrine signalling to protect alveolar integrity during abstinence of suckling.

There is an emerging awareness that multiple types of signals, that is, ECM proteins, soluble factors, physical forces and cell–cell interactions, control metazoan cell function. Deconvoluting how their intracellular effectors all interconnect to specify different fates will need both refined ways for analysing single cells and systems biology approaches.

## Integrins are checkpoints for GF signalling in normal breast and cancer

Integrins ‘integrate’ cells into their tissues by binding cells to the ECM and simultaneously organising the cytoskeleton (Figure 2c). To link to the cell interior, integrins, which have short non-enzymatic cytoplasmic tails, assemble large multi-protein machines at the plasma membrane [48]. The combined ability to bind ECM proteins and control cytoskeletal dynamics and signalling allow integrins to sense the physical and chemical nature of the microenvironment, and to adjust intracellular responses appropriately.

For example, integrins are checkpoints for the normal proliferation of mammary cells during development [11]. In culture models, β1-integrins control the outcome of EGF signalling, by activating a Rac1 signalling pathway that controls Erk nuclear import [49]. Integrins signal directly to cell cycle via the adhesion complex protein talin, which is necessary for Fak activation and p21 suppression, and thereby providing the conditions for GFs to drive cell cycle [50^••^].

The function of integrins as adhesion checkpoints for hormone and growth factor signalling is crucial for maintaining the normal architecture and integrity of mammary tissue [5]. Correspondingly, perturbations of the integrin axis contribute to the major pathology of breast, which is cancer. Too high levels of β1-integrins, β5-integrins or β6-integrins [51–54], or too much activity of integrin signalling components, co-operate with oncogenes to drive excessive GF signalling. For example, Fak, Src and small GTPase activators Trio, Vav3 and P-Rex1 are frequently upregulated in breast cancer [55–58]. This leads to increased epithelial proliferation, or reduced apoptosis in response to damage. Other elevated adhesion complex proteins, for example, kindlin-1, contribute to advanced breast disease, including metastasis [59^•^].

Integrin function can also alter in disease progression. α6β4-integrin is normally involved with cell adhesion to BM via hemidesmosomes and seems to be not essential for normal development [9,60]. However, in advanced breast carcinomas α6β4 relocates to the leading front of invasive cells, where it cooperates with ErbB2 and ErbB3 to promote inappropriate signalling [9,61].

Thus, as with the other classic cell cycle and apoptosis checkpoints that become deregulated in cancer, so do the integrin checkpoints. Integrins maintain tissue architecture and function in the normal breast, while disrupting the integrin axis alters signalling and causes tissue disorganisation and malignancy.

## Altered integrins cause tissue disorganisation and metastasis in breast cancer

An early event to arise during breast cancer initiation is tissue disorganisation (Figure 1c). The luminal cells that form the majority of breast cancers undergo massive bursts of proliferation each menstrual/oestrus cycle and during pregnancy. Epithelial proliferation is spatially orchestrated in normal breast, but what distinguishes this process from cancer is that the latter is disorganised. Moreover, tumours originate initially from single cells. This has been modelled in 3D cultured breast acini by using limiting dilutions of lentiviral infection [62]. Some breast oncogenes, for example, ErbB2, cause normal surrounding cells to extrude the rogue oncogene-expressing cell into the acinar lumen, where it proliferates abnormally. Other oncogenes require assistance from a second change, for example, oncogenic Akt1 requires MT1-MMP activation or talin depletion to cause rogue cell displacement. This argues that altered cell–matrix interactions may contribute to the early stages of cancer, by disrupting the normal spatial architecture of breast ducts and lobules.

In early breast hyperplasias and in situ carcinoma, epithelial cell masses remain encapsulated by myoepithelial cells and BM, but the cells loose polarity and the discrete bi-layered organisation. Microarray data has revealed that the expression of a variety of genes encoding cell–matrix proteins is altered during the progression of early breast cancers, including atypical ductal hyperplasia and ductal carcinoma in situ [63]. Although cell extrusion contributes to tissue disorganisation, loss of polarity also has a key role. Polarity genes are deregulated in early disease, and some, such as Par3, are potent metastasis suppressors [64,65]. Similarly, reduced levels of adhesion complex proteins that normally integrate breast epithelia within their tissue environment contribute to malignancy. For example the collagen receptor, α2β1-integrin, is a metastasis suppressor. Its levels are reduced in advanced breast cancer, and deleting the α2-integrin gene promotes metastases [66^•^]. Integrin signalling proteins such as nischarin and the Rho-GEFs Vav2/3 are further tumour suppressors in breast [15,16,67^•^,68].

The β3-integrins may contribute to tumour angiogenesis and progression of some classes of breast cancer, and although β3-integrins are expressed in mammary stem cells, their role in normal breast are not known. [69,70].

A gap in our knowledge is how levels of integrin signalling proteins change in breast cancer. Future work to dissect the impact of adhesion mutations on breast cancer, and a greater emphasis on the epigenetic control of cell–matrix adhesion is urgent, as this may provide valuable new strategies to tackle disease [71–74].

## The stromal microenvironment is a key player in breast cancer

**Figure 3:**
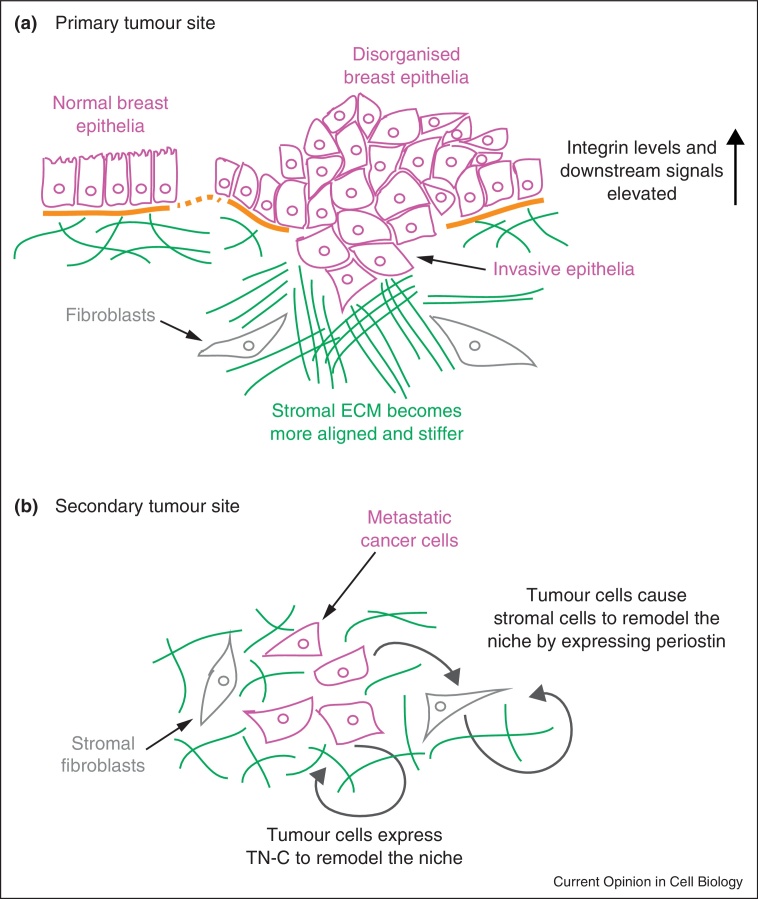
**(a)** Integrins and integrin signalling proteins are upregulated or activated at the primary breast tumour site. This leads to tissue disorganisation and loss of polarity. The stromal ECM changes, becoming stiffer. Together changes in integrin signalling promote increased proliferation, reduced apoptosis, and enhanced migration. **(b)** Tumour cells influence cell–ECM interactions at the secondary site by remodelling the stromal niche. This can occur in a variety of ways, including direct expression of proteins normally expressed in embryogenesis (tenascin), and indirect remodelling via stromal cell intermediates, for example in the expression of periostin.

Breast stroma has come to the fore as a major player in tumour development [75]. The tumour microenvironment promotes the deposition of ECM proteins into cross-linked fibers, whose alignment results in a stiffer stroma and poorer prognosis for cancer (Fig. 3a) [76^•^]. This contributes to tumorigenesis via integrin mechanosensing receptors, leading to enhanced Rho-mediated contractility and activation of integrin signalling. For example in stiff matrices, Fak promotes Mdm-2-dependent p53 degradation thereby preventing apoptosis [77]. These conditions also activate the Ras/Erk and PI3K pathways, further enhancing survival and proliferation [78].

ECM proteins that are abnormally expressed by infiltrating tumour or stromal cells promote invasion and metastasis. The laminin receptor, α6β4-integrin, regulates expression of SPARC (secreted protein acidic and rich in cysteine), which is involved with matrix remodelling [79]. SPARC is negatively controlled by miR-29a, and α6β4-integrin down-regulates this microRNA thereby enhancing SPARC expression and promoting invasion. Tenascin-C, often up-regulated in breast cancer, induces Fak/Src activation, partial EMT, enhanced migration, and quick progression to lung relapse [80]. Moreover, elevated Tenascin-C enhances expression of the stem cell regulators, musashi homolog 1 and leucine-rich repeat-containing G protein-coupled receptor 5, and promotes the growth of pulmonary micrometastases, while its knock-down diminishes lung metastases (Fig. 3b) [81^••^]. Finally, periostin is a stromal ECM protein that is abnormally expressed in the tumour microenvironment and in metastases [82]. To initiate lung metastases, breast cancer cells induce the expression of periostin in lung stroma [83^••^]. The establishment of a distant niche by stromal cells in response to paracrine tumour cell signalling appears to be an important step in metastasis formation.

These new findings about how the stroma contributes to breast cancer through altered expression of ECM proteins and assembly of ECM architecture, and how tumour cells prime the metastatic niche, profoundly change the way we think about cancer progression.

## Integrin-targeted therapies for breast cancer

Taken together, integrins have a central role in regulating all aspects of mammary gland function, and perturbed integrin signalling is required for breast cancer to progress (Figure 3). The ECM–integrin axis is therefore a promising target for combatting disease [84]. Although hormone and GF antagonists are effective for some types of cancer, the more intractable cancers, for example, triple-negative malignant lesions, remain incurable. More emphasis on intervening with cell–matrix interactions will considerably augment current treatments, by targeting the TIC niche and metastases.

Some integrin-directed approaches are in pre-clinical trials. For example, anti-integrin antibodies, in combination with radiotherapy, may be showing early signs of success [85,86]. Moreover, many integrin antagonists are currently under clinical development [87]. Small molecules such as cilengitide (a cyclic anti-integrin RGD mimetic) may reduce metastatic colonisation and inhibiting bone resorption in established metastases [88,89]. Particularly exciting are the new combinatorial approaches to target cancer stromal cells: combined antagonists of monocyte α4β1 integrin and SDFα or IL-1β inhibit tumour inflammation and growth [90], and a recombinant protein jointly targeting integrins and VEGF is an effective inhibitor of breast cancer angiogenesis [91].

However, although some of these strategies target cancer cells themselves, careful use of therapies will be needed in the light of integrin's ability to both promote cancer and to act as tumour suppressors.

## References and recommended reading

Papers of particular interest, published within the period of review, have been highlighted as:

• of special interest
•• of outstanding interest

## References

[1] Muschler J., Streuli C.H. (2010). Cell–matrix interactions in mammary gland development and breast cancer. Cold Spring Harb Perspect Biol.

[2] Lu P., Weaver V.M., Werb Z. (2012). The extracellular matrix: a dynamic niche in cancer progression. J Cell Biol.

[3] Raymond K., Faraldo M.M., Deugnier M.-A., Glukhova M.A. (2012). Integrins in mammary development. Semin Cell Dev Biol.

[4] Streuli C.H. (2009). Integrins and cell-fate determination. J Cell Sci.

[5] Katz E., Streuli C.H. (2007). The extracellular matrix as an adhesion checkpoint for mammary epithelial function. Int J Biochem Cell Biol.

[6] Eyckmans J., Boudou T., Yu X., Chen C.S. (2011). A hitchhiker's guide to mechanobiology. Dev Cell.

[7•] Huck L., Pontier S.M., Zuo D.M., Muller W.J. (2010). beta1-integrin is dispensable for the induction of ErbB2 mammary tumors but plays a critical role in the metastatic phase of tumor progression. Proc Natl Acad Sci USA.

[8] White D.E., Kurpios N.A., Zuo D., Hassell J.A., Blaess S., Mueller U., Muller W.J. (2004). Targeted disruption of beta1-integrin in a transgenic mouse model of human breast cancer reveals an essential role in mammary tumor induction. Cancer Cell.

[9] Guo W., Pylayeva Y., Pepe A., Yoshioka T., Muller W.J., Inghirami G., Giancotti F.G. (2006). Beta 4 integrin amplifies ErbB2 signaling to promote mammary tumorigenesis. Cell.

[10] Van Keymeulen A., Rocha A.S., Ousset M., Beck B., Bouvencourt G., Rock J., Sharma N., Dekoninck S., Blanpain C. (2011). Distinct stem cells contribute to mammary gland development and maintenance. Nature.

[11] Li N., Zhang Y., Naylor M.J., Schatzmann F., Maurer F., Wintermantel T., Schuetz G., Mueller U., Streuli C.H., Hynes N.E. (2005). Beta1 integrins regulate mammary gland proliferation and maintain the integrity of mammary alveoli. EMBO J.

[12••] Taddei I., Deugnier M.-A., Faraldo M.M., Petit V., Bouvard D., Medina D., Fässler R., Thiery J.P., Glukhova M.A. (2008). Beta1 integrin deletion from the basal compartment of the mammary epithelium affects stem cells [Internet]. Nat Cell Biol.

[13] Smalley M.J., Kendrick H., Sheridan J.M., Regan J.L., Prater M.D., Lindeman G.J., Watson C.J., Visvader J.E., Stingl J. (2012). Isolation of mouse mammary epithelial subpopulations: a comparison of leading methods. J Mammary Gland Biol Neoplasia.

[14] Shehata M., Teschendorff A., Sharp G., Novcic N., Russell I.A., Avril S., Prater M., Eirew P., Caldas C., Watson C.J. (2012). Phenotypic and functional characterisation of the luminal cell hierarchy of the mammary gland. Breast Cancer Res.

[15] Vaillant F., Asselin-Labat M.-L., Shackleton M., Forrest N.C., Lindeman G.J., Visvader J.E. (2008). The mammary progenitor marker CD61/beta3 integrin identifies cancer stem cells in mouse models of mammary tumorigenesis. Cancer Res.

[16•] Lo P.-K., Kanojia D., Liu X., Singh U.P., Berger F.G., Wang Q., Chen H. (2012). CD49f and CD61 identify Her2/neu-induced mammary tumor-initiating cells that are potentially derived from luminal progenitors and maintained by the integrin-TGFβ signaling. Oncogene.

[17] Cariati M., Naderi A., Brown J.P., Smalley M.J., Pinder S.E., Caldas C., Purushotham A.D. (2008). Alpha-6 integrin is necessary for the tumourigenicity of a stem cell-like subpopulation within the MCF7 breast cancer cell line. Int J Cancer.

[18] Gilmore A.P., Metcalfe A.D., Romer L.H., Streuli C.H. (2000). Integrin-mediated survival signals regulate the apoptotic function of Bax through its conformation and subcellular localization. J Cell Biol.

[19••] Schellenberg B., Wang P., Keeble J.A., Rodriguez-Enriquez R., Walker S., Owens T.W., Foster F., Tanianis-Hughes J., Brennan K., Streuli C.H. (2013). Bax exists in a dynamic equilibrium between the cytosol and mitochondria to control apoptotic priming. Mol Cell.

[20] Cicalese A., Bonizzi G., Pasi C.E., Faretta M., Ronzoni S., Giulini B., Brisken C., Minucci S., Di Fiore P.P., Pelicci P.G. (2009). The tumor suppressor p53 regulates polarity of self-renewing divisions in mammary stem cells. Cell.

[21] Luo M., Fan H., Nagy T., Wei H., Wang C., Liu S., Wicha M.S., Guan J.-L. (2009). Mammary epithelial-specific ablation of the focal adhesion kinase suppresses mammary tumorigenesis by affecting mammary cancer stem/progenitor cells. Cancer Res.

[22] Goel H.L., Pursell B., Standley C., Fogarty K., Mercurio A.M. (2012). Neuropilin-2 regulates α6β1 integrin in the formation of focal adhesions and signaling. J Cell Sci.

[23•] Goel H.L., Pursell B., Chang C., Shaw L.M., Mao J., Simin K., Kumar P., Kooi C.W.V., Shultz L.D., Greiner D.L. (2013). GLI1 regulates a novel neuropilin-2/α6β1 integrin based autocrine pathway that contributes to breast cancer initiation. EMBO Mol Med.

[24] Hinck L., Silberstein G.B. (2005). Key stages in mammary gland development: the mammary end bud as a motile organ. Breast Cancer Res.

[25] Klinowska T.C., Soriano J.V., Edwards G.M., Oliver J.M., Valentijn A.J., Montesano R., Streuli C.H. (1999). Laminin and beta1 integrins are crucial for normal mammary gland development in the mouse. Dev Biol.

[26] Lu P., Ewald A.J., Martin G.R., Werb Z. (2008). Genetic mosaic analysis reveals FGF receptor 2 function in terminal end buds during mammary gland branching morphogenesis. Dev Biol.

[27•] Ewald A.J., Brenot A., Duong M., Chan B.S., Werb Z. (2008). Collective epithelial migration and cell rearrangements drive mammary branching morphogenesis. Dev Cell.

[28••] Brownfield D.G., Venugopalan G., Lo A., Mori H., Tanner K., Fletcher D.A., Bissell M.J. (2013). Patterned Collagen fibers orient branching mammary epithelium through distinct signaling modules. Current Biology.

[29] Sakakura T., Nishizuka Y., Dawe C.J. (1976). Mesenchyme-dependent morphogenesis and epithelium-specific cytodifferentiation in mouse mammary gland. Science.

[30] Onodera T., Sakai T., Hsu J.C., Matsumoto K., Chiorini J.A., Yamada K.M. (2010). Btbd7 regulates epithelial cell dynamics and branching morphogenesis. Science.

[31] Daley W.P., Kohn J.M., Larsen M. (2011). A focal adhesion protein-based mechanochemical checkpoint regulates cleft progression during branching morphogenesis. Dev Dyn.

[32] Rebustini I.T., Myers C., Lassiter K.S., Surmak A., Szabova L., Holmbeck K., Pedchenko V., Hudson B.G., Hoffman M.P. (2009). MT2-MMP-dependent release of collagen IV NC1 domains regulates submandibular gland branching morphogenesis. Dev Cell.

[33•] Mori H., Lo A.T., Inman J.L., Alcaraz J., Ghajar C.M., Mott J.D., Nelson C.M., Chen C.S., Zhang H., Bascom J.L. (2013). Transmembrane/cytoplasmic, rather than catalytic, domains of Mmp14 signal to MAPK activation and mammary branching morphogenesis via binding to integrin β1. Development.

[34•] Nguyen-Ngoc K.-V., Cheung K.J., Brenot A., Shamir E.R., Gray R.S., Hines W.C., Yaswen P., Werb Z., Ewald A.J. (2012). ECM microenvironment regulates collective migration and local dissemination in normal and malignant mammary epithelium. Proc Natl Acad Sci USA.

[35] Yoshida T., Matsumoto E., Hanamura N., Kalembeyi I., Katsuta K., Ishihara A., Sakakura T. (1997). Co-expression of tenascin and fibronectin in epithelial and stromal cells of benign lesions and ductal carcinomas in the human breast. J Pathol.

[36••] Raymond K., Cagnet S., Kreft M., Janssen H., Sonnenberg A., Glukhova M.A. (2011). Control of mammary myoepithelial cell contractile function by α3β1 integrin signalling. EMBO J.

[37••] Akhtar N., Streuli C.H. (2013). An integrin-ILK-microtubule network orients cell polarity and lumen formation in glandular epithelium. Nat Cell Biol.

[38] Wickström S.A., Lange A., Hess M.W., Polleux J., Spatz J.P., Krüger M., Pfaller K., Lambacher A., Bloch W., Mann M. (2010). Integrin-linked kinase controls microtubule dynamics required for plasma membrane targeting of caveolae. Dev Cell.

[39•] Hotta A., Kawakatsu T., Nakatani T., Sato T., Matsui C., Sukezane T., Akagi T., Hamaji T., Grigoriev I., Akhmanova A. (2010). Laminin-based cell adhesion anchors microtubule plus ends to the epithelial cell basal cortex through LL5alpha/beta. J Cell Biol.

[40•] Ahmed S.M., Thériault B.L., Uppalapati M., Chiu C.W.N., Gallie B.L., Sidhu S.S., Angers S. (2012). KIF14 negatively regulates Rap1a-Radil signaling during breast cancer progression. J Cell Biol.

[41] Naylor M.J., Li N., Cheung J., Lowe E.T., Lambert E., Marlow R., Wang P., Schatzmann F., Wintermantel T., Schuetz G. (2005). Ablation of beta1 integrin in mammary epithelium reveals a key role for integrin in glandular morphogenesis and differentiation. J Cell Biol.

[42] Akhtar N., Streuli C.H. (2006). Rac1 links integrin-mediated adhesion to the control of lactational differentiation in mammary epithelia. J Cell Biol.

[43] Akhtar N., Marlow R., Lambert E., Schatzmann F., Lowe E.T., Cheung J., Katz E., Li W., Wu C., Dedhar S. (2009). Molecular dissection of integrin signalling proteins in the control of mammary epithelial development and differentiation. Development.

[44] Edwards G.M., Wilford F.H., Liu X., Hennighausen L., Djiane J., Streuli C.H. (1998). Regulation of mammary differentiation by extracellular matrix involves protein-tyrosine phosphatases. J Biol Chem.

[45] Zoubiane G.S., Valentijn A., Lowe E.T., Akhtar N., Bagley S., Gilmore A.P., Streuli C.H. (2004). A role for the cytoskeleton in prolactin-dependent mammary epithelial cell differentiation. J Cell Sci.

[46•] Du J.-Y., Chen M.-C., Hsu T.-C., Wang J.-H., Brackenbury L., Lin T.-H., Wu Y.-Y., Yang Z., Streuli C.H., Lee Y.-J. (2012). The RhoA-Rok-myosin II pathway is involved in extracellular matrix-mediated regulation of prolactin signaling in mammary epithelial cells. J Cell Physiol.

[47] Mammoto A., Mammoto T., Ingber D.E. (2012). Mechanosensitive mechanisms in transcriptional regulation. J Cell Sci.

[48] Streuli C.H., Akhtar N. (2009). Signal co-operation between integrins and other receptor systems. Biochem J.

[49] Jeanes A.I., Wang P., Moreno-Layseca P., Paul N., Cheung J., Tsang R., Akhtar N., Foster F.M., Brennan K., Streuli C.H. (2012). Specific β-containing integrins exert differential control on proliferation and two-dimensional collective cell migration in mammary epithelial cells. J Biol Chem.

[50••] Wang P., Ballestrem C., Streuli C.H. (2011). The C terminus of talin links integrins to cell cycle progression. J Cell Biol.

[51] Dos Santos P.B., Zanetti J.S., Ribeiro-Silva A., Beltrao E.I. (2012). Beta 1 integrin predicts survival in breast cancer: a clinicopathological and immunohistochemical study. Diagn Pathol.

[52] Yao E.S., Zhang H., Chen Y.-Y., Lee B., Chew K., Moore D., Park C. (2007). Increased beta1 integrin is associated with decreased survival in invasive breast cancer. Cancer Res.

[53] Bianchi-Smiraglia A., Paesante S., Bakin A.V. (2012). Integrin β5 contributes to the tumorigenic potential of breast cancer cells through the Src-FAK and MEK-ERK signaling pathways. Oncogene.

[54] Goodman S.L., Grote H.J., Wilm C. (2012). Matched rabbit monoclonal antibodies against αv-series integrins reveal a novel αvβ3-LIBS epitope, and permit routine staining of archival paraffin samples of human tumors. Biol Open.

[55] Lane J., Martin T.A., Mansel R.E., Jiang W.G. (2008). The expression and prognostic value of the guanine nucleotide exchange factors (GEFs) Trio, Vav1 and TIAM-1 in human breast cancer. Int Seminars Surg Oncol.

[56] Lee K., Liu Y., Mo J.Q., Zhang J., Dong Z., Lu S. (2008). Vav3 oncogene activates estrogen receptor and its overexpression may be involved in human breast cancer. BMC Cancer.

[57] Sosa M.S., Lopez-Haber C., Yang C., Wang H., Lemmon M.A., Busillo J.M., Luo J., Benovic J.L., Klein-Szanto A., Yagi H. (2010). Identification of the Rac-GEF P-Rex1 as an essential mediator of ErbB signaling in breast cancer. Mol Cell.

[58] Zouq N.K., Keeble J.A., Lindsay J., Valentijn A.J., Zhang L., Mills D., Turner C.E., Streuli C.H., Gilmore A.P. (2009). FAK engages multiple pathways to maintain survival of fibroblasts and epithelia: differential roles for paxillin and p130Cas. J Cell Sci.

[59•] Sin S., Bonin F., Petit V., Meseure D., Lallemand F., Bièche I., Bellahcène A., Castronovo V., De Wever O., Gespach C. (2011). Role of the focal adhesion protein kindlin-1 in breast cancer growth and lung metastasis. J Natl Cancer Inst.

[60] Klinowska T.C., Alexander C.M., Georges-Labouesse E., Van der Neut R., Kreidberg J.A., Jones C.J., Sonnenberg A., Streuli C.H. (2001). Epithelial development and differentiation in the mammary gland is not dependent on alpha 3 or alpha 6 integrin subunits. Dev Biol.

[61] Lu S., Simin K., Khan A., Mercurio A.M. (2008). Analysis of integrin beta4 expression in human breast cancer: association with basal-like tumors and prognostic significance. Clin Cancer Res.

[62] Leung C.T., Brugge J.S. (2012). Outgrowth of single oncogene-expressing cells from suppressive epithelial environments. Nature.

[63] Emery L.A., Tripathi A., King C., Kavanah M., Mendez J., Stone M.D., De las Morenas A., Sebastiani P., Rosenberg C.L. (2009). Early dysregulation of cell adhesion and extracellular matrix pathways in breast cancer progression. Am J Pathol.

[64] McCaffrey L.M., Montalbano J., Mihai C., Macara I.G. (2012). Loss of the Par3 polarity protein promotes breast tumorigenesis and metastasis. Cancer Cell.

[65] Xue B., Krishnamurthy K., Allred D.C., Muthuswamy S.K. (2012). Loss of Par3 promotes breast cancer metastasis by compromising cell-cell cohesion. Nat Cell Biol.

[66•] Ramirez N.E., Zhang Z., Madamanchi A., Boyd K.L., O’Rear L.D., Nashabi A., Li Z., Dupont W.D., Zijlstra A., Zutter M.M. (2011). The α_2_β_1_ integrin is a metastasis suppressor in mouse models and human cancer. J Clin Invest.

[67•] Baranwal S., Wang Y., Rathinam R., Lee J., Jin L., McGoey R., Pylayeva Y., Giancotti F., Blobe G.C., Alahari S.K. (2011). Molecular characterization of the tumor-suppressive function of nischarin in breast cancer. J Natl Cancer Inst.

[68] Citterio C., Menacho-Márquez M., García-Escudero R., Larive R.M., Barreiro O., Sánchez-Madrid F., Paramio J.M., Bustelo X.R. (2012). The rho exchange factors vav2 and vav3 control a lung metastasis-specific transcriptional program in breast cancer cells. Sci Signal.

[69] Robinson S.D., Hodivala-Dilke K.M. (2011). The role of β3-integrins in tumor angiogenesis: context is everything. Curr Opin Cell Biol.

[70] Taverna D., Crowley D., Connolly M., Bronson R.T., Hynes R.O. (2005). A direct test of potential roles for beta3 and beta5 integrins in growth and metastasis of murine mammary carcinomas. Cancer Res.

[71] Yang X., Pursell B., Lu S., Chang T.-K., Mercurio A.M. (2009). Regulation of beta 4-integrin expression by epigenetic modifications in the mammary gland and during the epithelial-to-mesenchymal transition. J Cell Sci.

[72] Mostovich L.A., Prudnikova T.Y., Kondratov A.G., Loginova D., Vavilov P.V., Rykova V.I., Sidorov S.V., Pavlova T.V., Kashuba V.I., Zabarovsky E.R. (2011). Integrin alpha9 (ITGA9) expression and epigenetic silencing in human breast tumors. Cell Adh Migr.

[73] Mulder K.W., Wang X., Escriu C., Ito Y., Schwarz R.F., Gillis J., Sirokmány G., Donati G., Uribe-Lewis S., Pavlidis P. (2012). Diverse epigenetic strategies interact to control epidermal differentiation. Nat Cell Biol.

[74] Shah S.P., Roth A., Goya R., Oloumi A., Ha G., Zhao Y., Turashvili G., Ding J., Tse K., Haffari G. (2012). The clonal and mutational evolution spectrum of primary triple-negative breast cancers. Nature.

[75] DuFort C.C., Paszek M.J., Weaver V.M. (2011). Balancing forces: architectural control of mechanotransduction. Nat Rev Mol Cell Biol.

[76•] Conklin M.W., Eickhoff J.C., Riching K.M., Pehlke C.A., Eliceiri K.W., Provenzano P.P., Friedl A., Keely P.J. (2011). Aligned collagen is a prognostic signature for survival in human breast carcinoma. Am J Pathol.

[77] Lim S.-T., Chen X.L., Lim Y., Hanson D.A., Vo T.-T., Howerton K., Larocque N., Fisher S.J., Schlaepfer D.D., Ilic D: Nuclear FAK (2008). promotes cell proliferation and survival through FERM-enhanced p53 degradation. Mol Cell.

[78] Provenzano P.P., Keely P.J. (2011). Mechanical signaling through the cytoskeleton regulates cell proliferation by coordinated focal adhesion and Rho GTPase signaling. J Cell Sci.

[79] Gerson K.D., Shearstone J.R., Maddula V.S.R.K., Seligmann B.E., Mercurio A.M. (2012). Integrin β4 regulates SPARC protein to promote invasion. J Biol Chem.

[80] Nagaharu K., Zhang X., Yoshida T., Katoh D., Hanamura N., Kozuka Y., Ogawa T., Shiraishi T., Imanaka-Yoshida K. (2011). Tenascin C induces epithelial–mesenchymal transition-like change accompanied by SRC activation and focal adhesion kinase phosphorylation in human breast cancer cells. Am J Pathol.

[81••] Oskarsson T., Acharyya S., Zhang X.H.-F., Vanharanta S., Tavazoie S.F., Morris P.G., Downey R.J., Manova-Todorova K., Brogi E., Massagué J. (2011). Breast cancer cells produce tenascin C as a metastatic niche component to colonize the lungs. Nat Med.

[82] Kharaishvili G., Cizkova M., Bouchalova K., Mgebrishvili G., Kolar Z., Bouchal J. (2011). Collagen triple helix repeat containing 1 protein, periostin and versican in primary and metastatic breast cancer: an immunohistochemical study. J Clin Pathol.

[83••] Malanchi I., Santamaria-Martínez A., Susanto E., Peng H., Lehr H.-A., Delaloye J.-F., Huelsken J. (2012). Interactions between cancer stem cells and their niche govern metastatic colonization. Nature.

[84] Barkan D., Chambers A.F. (2011). β1-integrin: a potential therapeutic target in the battle against cancer recurrence. Clin Cancer Res.

[85] Nam J.-M., Onodera Y., Bissell M.J., Park C.C. (2010). Breast cancer cells in three-dimensional culture display an enhanced radioresponse after coordinate targeting of integrin alpha5beta1 and fibronectin. Cancer Res.

[86] Park C.C., Zhang H.J., Yao E.S., Park C.J., Bissell M.J. (2008). Beta1 integrin inhibition dramatically enhances radiotherapy efficacy in human breast cancer xenografts [Internet]. Cancer Res.

[87] Goodman S.L., Picard M. (2012). Integrins as therapeutic targets. Trends Pharmacol Sci.

[88] Bretschi M., Merz M., Komljenovic D., Berger M.R., Semmler W., Bäuerle T. (2011). Cilengitide inhibits metastatic bone colonization in a nude rat model. Oncol Rep.

[89] Bäuerle T., Komljenovic D., Merz M., Berger M.R., Goodman S.L., Semmler W. (2011). Cilengitide inhibits progression of experimental breast cancer bone metastases as imaged noninvasively using VCT, MRI and DCE-MRI in a longitudinal in vivo study. Int J Cancer.

[90] Schmid M.C., Avraamides C.J., Foubert P., Shaked Y., Kang S.W., Kerbel R.S., Varner J.A. (2011). Combined blockade of integrin-α4β1 plus cytokines SDF-1α or IL-1β potently inhibits tumor inflammation and growth. Cancer Res.

[91] Wu J., Jiang Y., Yang W., He Z., Meng S., Zhang Q., Lin M., Zhang H., Li W., Yang Y. (2012). Dual function of RGD-modified VEGI-192 for breast cancer treatment. Bioconjug Chem.

